# Effects of the COVID-19 pandemic on patients with inflammatory joint diseases in Sweden: from infection severity to impact on care provision

**DOI:** 10.1136/rmdopen-2021-001987

**Published:** 2021-12-07

**Authors:** Hannah Bower, Thomas Frisell, Daniela di Giuseppe, Bénédicte Delcoigne, Gerd-Marie Alenius, Eva Baecklund, Katerina Chatzidionysiou, Nils Feltelius, Helena Forsblad-d'Elia, Alf Kastbom, Lars Klareskog, Elisabet Lindqvist, Ulf Lindström, Carl Turesson, Christopher Sjöwall, Johan Askling, The ARTIS Study Group, Gerd-Marie Ahlenius

**Affiliations:** 1 Clinical Epidemiology Division, Department of Medicine Solna, Karolinska Institutet, Stockholm, Sweden; 2 Department of Public Health and Clinical Medicine/Rheumatology, Umeå Universitet, Umeå, Sweden; 3 Unit of Rheumatology, Department of Medical Sciences, Uppsala University, Uppsala, Sweden; 4 Department of Medicine Solna, Karolinska Institutet, Stockholm, Sweden; 5 Swedish Medical Products Agency, Uppsala, Sweden; 6 Department of Public Health and Caring Sciences, Uppsala Universitet, Uppsala, Sweden; 7 Department of Rheumatology and Inflammation Research, Institute of Medicine, Sahlgrenska Academy, University of Gothenburg, Gothenburg, Sweden; 8 Department of Biomedical and Clinical Sciences, Linkopings Universitet, Linkoping, Sweden; 9 Department of Clinical Sciences, Rheumatology, Lund University, Lund, Sweden; 10 Rheumatology, Department of Clinical Sciences, Lund University, Malmö, Sweden; 11 Department of Biomedical and Clinical Sciences, Linköping University, Linkoping, Sweden

**Keywords:** rheumatoid arthritis, COVID-19, treatment, antirheumatic agents

## Abstract

**Objectives:**

To compare risks for COVID-19-related outcomes in inflammatory joint diseases (IJDs) and across disease-modifying antirheumatic drugs (DMARDs) during the first two waves of the pandemic and to assess effects of the pandemic on rheumatology care provision.

**Methods:**

Through nationwide multiregister linkages and cohort study design, we defined IJD and DMARD use annually in 2015–2020. We assessed absolute and relative risks of hospitalisation or death listing COVID-19. We also assessed the incidence of IJD and among individuals with IJD, rheumatologist visits, DMARD use and incidence of selected comorbidities.

**Results:**

Based on 115 317 patients with IJD in 2020, crude risks of hospitalisation and death listing COVID-19 (0.94% and 0.33% across both waves, respectively) were similar during both waves (adjusted HR versus the general population 1.33, 95% CI 1.23 to 1.43, for hospitalisation listing COVID-19; 1.23, 95% CI 1.08 to 1.40 for death listing COVID-19). Overall, biological disease-modifying antirheumatic drugs (bDMARDs)/targeted synthetic disease-modifying antirheumatic drugs (tsDMARDs) did not increase risks of COVID-19 related hospitalisation (with the exception of a potential signal for JAK inhibitors) or death. During the pandemic, decreases were observed for IJD incidence (−7%), visits to rheumatology units (−16%), DMARD dispensations (+6.5% for bDMARD/tsDMARDs and −8.5% for conventional synthetic DMARDs compared with previous years) and for new comorbid conditions, but several of these changes were part of underlying secular trends.

**Conclusions:**

Patients with IJD are at increased risk of serious COVID-19 outcomes, which may partially be explained by medical conditions other than IJD per se. The SARS-CoV-2 pandemic has exerted measurable effects on aspects of rheumatology care provision demonstrated, the future impact of which will need to be assessed.

**Key messages**

**What is already known about this subject?**

- Studies, predominantly from the first wave of the COVID-19 pandemic, have reported that patients with inflammatory joint diseases (IJDs) are at increased risks of serious outcomes of SARS-CoV-2 infection.
- Survey studies during the same time have also indicated marked changes in the (attitudes towards) use of antirheumatic drugs and visits to rheumatologists.

**What does this study add?**

- Patients with IJDs are at increased risk of hospitalisation or death from COVID-19, equally so during the second as during the first wave. This increased risk may be explained by medical conditions other than the rheumatic disease per se; common antirheumatic treatments do not seem to increase this risk (with the potential exception of JAK inhibitors and rituximab).
- The effects of the pandemic on care provision for IJDs have been modest when considering yearly trends.

**How might this impact on clinical practice or future developments?**

- Our study highlights the importance of putting changes during the peak of a pandemic wave into context (eg, height of pandemic versus entire calendar year and versus underlying trends).
- Annual changes in care provision for IJDs during the pandemic are in many (but not all) respects smaller than annual changes from underlying secular trends.
- The consequences of changes in care provision of IJD observed will need to be monitored.

## Introduction

The COVID-19 pandemic hit patients with inflammatory joint diseases (IJDs) both directly and indirectly. In terms of direct impact, studies have demonstrated increased risks of serious COVID-19 in patients with IJD compared with the general population.^1–4^ Most of these studies are based on data from the first ‘wave’ of the COVID-19 pandemic. The second wave was more penetrating in terms of number of confirmed cases and deaths, but also came with improved in-hospital care including the use of corticosteroids and optimised use of oxygen, which may have affected case fatality.^5^ Additionally, attitudes and behaviours surrounding COVID-19 may have changed from early spring 2020. Since first-wave experiences are thus not necessarily applicable to later waves, and since signals of increased risks associated with certain disease-modifying antirheumatic drugs (DMARDs)^1 6 7^ have often been based on limited statistical precision and data with uncertain generalisability, more data on outcomes of SARS-COV-2 infection in IJD are needed.

In terms of indirect impact, public health measures instituted to limit the spread of the COVID-19 pandemic have greatly affected the provision of care^8^ and may have resulted in patients’ and doctors’ delay in diagnosing new-onset IJD. Similarly, reluctance to continue ongoing, or to start new, DMARDs may have negatively affected the level of disease control in IJD. Changes in visit patterns may have affected not only the means to identify diagnosis and treatment of IJD, but also the timely detection of comorbid conditions in IJD. In the general population, profound declines in the observed incidence of, for example, breast cancer and myocardial infarction (MI) during the first wave of the pandemic have been reported.^9 10^ All of the aforementioned effects may have important long-term consequences, beyond the pandemic, and need to be assessed.

The aims of this study were therefore twofold: (1) to extend our assessment of outcomes of COVID-19 in patients with IJD to also encompass the second wave of the pandemic and to more precisely assess the impact of DMARDs on these outcomes, and (2) to assess the impact of the SARS-CoV-2 pandemic on the provision of rheumatology care, measured as the number of diagnoses for new-onset IJD, visit and treatment patterns among individuals with IJD, and occurrence of cancer and MI in patients with IJD, during the first and second waves of the pandemic and compared with previous years.

## Methods

### Setting

Swedish healthcare is public and tax-funded. Patients with IJDs treated with DMARDs are managed by rheumatologists, mainly through hospital-based clinics. The COVID-19 pandemic hit Sweden with a first wave between March and June 2020, and a second wave between October 2020 and Spring 2021. Throughout the pandemic, Sweden has had a relatively high incidence of infection and a mortality rate close to the European median.^11^ Recommendations from the Swedish Public Health Agency (not legally binding) has urged social distancing when possible, in particular for risk groups and those aged above 70. During the second wave, public health measures included (early) closure of restaurants, a cap on public gatherings and working/schooling remotely. No specific recommendations have been in place for IJD.

### Data sources

We updated an existing multiregister linkage (Anti-Rheumatic Therapies in Sweden, ‘ARTIS’)^12^ across national Swedish registers (online supplemental table S1) to cover follow-up data through 31 January 2021, that is, through most of the second wave.

10.1136/rmdopen-2021-001987.supp1 Supplementary data

### Study population

Previously,^1^ we defined a cohort of adult patients with IJD from inpatient and outpatient visits recorded in the National Patient Register (NPR) for the following conditions: rheumatoid arthritis (RA), psoriatic arthritis, ankylosing spondylitis, other spondyloarthropathies or juvenile idiopathic arthritis (online supplemental table S2). To define conventional synthetic disease-modifying antirheumatic drug (csDMARD) and biological disease-modifying antirheumatic drug (bDMARD)/targeted synthetic disease-modifying antirheumatic drug (tsDMARD) treatment, we used information from the Swedish Rheumatology Quality Register and the Prescribed Drug Register (PDR, online supplemental table S2). Patient and treatment cohorts for wave-specific analyses were selected based on their disease and DMARD status on 1 March (wave 1) and 1 October (wave 2) 2020.

For each unique individual with IJD, we randomly selected five individuals from the general Swedish population, matched on sex, year of birth and region of domicile, the year when the index individual with IJD was first registered with IJD.

### Part 1: outcomes of COVID-19 during the first and second waves

We extended follow-up of our previous report on COVID-19 outcomes during the first wave to also include the second wave.^1^ In brief, the outcomes hospitalisation listing COVID-19 and death due to COVID-19 in the IJD and in the comparator populations during March–June 2020 and during October 2020–31 January 2021 were identified using the NPR and Cause of Death Register (online supplemental table S3). We followed individuals from cohort entry to the event of interest, or censoring at death, migration from Sweden or end of follow-up (wave-dependent). We used register linkages to identify relevant covariates: age, sex, region of domicile, characteristics of the IJD including Disease Activity Score on 28 joints (DAS28) and disease duration, prevalence of comorbidities and history of hospitalisations, educational level, country of birth and civil status (online supplemental table S4).

For each outcome and wave and for the two waves combined, we calculated crude incidence rates, and relative risks estimated as HR comparing IJD to the general population through Cox regression. HRs were further adjusted for history of comorbidities and socioeconomic factors.

We next determined the DMARD treatment status in the IJD cohort at the beginning of the first and the second waves (online supplemental table S2), identified previous bDMARDs, concomitant csDMARD and steroid use through additional linkages, and calculated crude risks for each outcome and drug category. We used inverse probability of treatment-weighted (IPTW) Cox regression to estimate HRs comparing bDMARD/tsDMARDs to csDMARDs, accommodating for age, sex, region, characteristics of IJD, comorbidities and socioeconomic factors in the IPTW, and additionally adjusting for previous bDMARD/tsDMARDs, concomitant csDMARD or steroids (online supplemental materials). Wald tests were performed to test whether incidences and HRs differed between waves. No imputation of missing data was performed.

### Part 2: effects of the COVID-19 pandemic on care provision for IJDs

#### Observed incidence of IJDs

For each year from 2015 to 2020, we assessed incidence rates of newly diagnosed IJD among adults in Sweden. IJD incidence was defined as a first registration with any of the IJD-defining conditions in the NPR in individuals without a history of IJD, divided by the monthly Swedish population size above 18 years.^13^

#### Visits in specialised care

For all at-risk individuals with IJD at the beginning of each month from 2015 to 2020, we calculated the rate of visits (for any diagnosis and for IJD, respectively) to a department of rheumatology or internal medicine. We plotted monthly visit incidences overlaid (per year) and separately by IJD type (RA vs all other IJD), IJD disease duration (less than vs at least 1 year), attained age (less than vs at least 70 years) and visit diagnosis (IJD vs any other ICD code). Finally, we calculated and plotted the monthly visit incidence during 2020 as a proportion of the corresponding average from 2015 to 2019.

#### Use of DMARDs

We similarly assessed DMARD treatment dispensations, treatment starts and stops during each month 2020, and during 2015–2019. We used a predetermined algorithm (online supplemental table S3) applied to data from the PDR to define DMARD categories and start and stop dates. We calculated the proportion of treatment dispensations, starts and stops per IJD individuals, per month, and plotted these.

#### Occurrence of diagnosed comorbidities

For each IJD cohort (2020 and 2015–2019) and for their matched general population comparator subjects, we calculated the incidence of first invasive malignancy (other than non-melanoma skin cancer) and first acute MI (online supplemental table S3) in individuals without a history of the condition over each year, presented as the proportion of individuals in each cohort every calendar month.

## Results

### Part 1: outcomes of SARS-CoV-2 infection during the first and second waves

**Figure 1:**
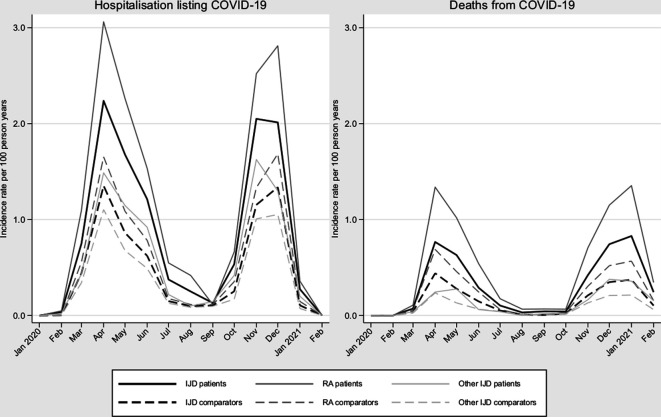
Crude incidence rates of hospitalisation listing COVID-19 and death due to COVID-19 in patients with IJD and their individually matched general population comparator subjects, from January 2020 until 31 January 2021. IJD, inflammatory joint disease; RA, rheumatoid arthritis.

Figure 1 presents crude rates of hospitalisation and death listing COVID-19 between 1 January 2020 until 31 January 2021. As previously reported, these rates were increased in IJD during March–June 2020.^1^ During the second wave (here: October 2020–January 2021), the absolute and relative risks were similar to the first wave, with absolute risks of 0.49% and 0.40% for hospitalisation listing COVID-19 during waves 1 and 2, and 0.15% and 0.17% for deaths from COVID-19, respectively (table 1). Compared with the general population, the adjusted HRs for IJD were similar during the first and second waves (table 1, Wald test for interaction p=0.59 and 0.57 for hospitalisation and death listing COVID-19, respectively). The crude HR for hospitalisation listing COVID-19 (both waves combined) was 1.78 (95% CI 1.66 to 1.91), which decreased to 1.33 (95% CI 1.23 to 1.43) following adjustment for the predefined comorbidities and socioeconomic factors. Similarly, the crude HR for death listing COVID-19 was 2.07 (95% CI 1.83 to 2.33), which decreased to 1.23 (95% CI 1.08 to 1.40) following adjustment (table 1).

**Table 1:** Events, risk, and crude incidence rate for patients with IJD and matched general population comparator subjects, alongside HRs and 95% CIs comparing IJD to matched general population comparator subjects for outcomes hospitalisation listing COVID-19 and death from COVID-19

		Events (N), risk (%)		Crude incidence rate, per 100 person-years		Unadjusted HR*	Adjusted HR†
		IJD	Comparators	IJD	Comparators		
IJD							
Hospitalisation, COVID-19	Overall	1090 (0.94)	2735 (0.53)	0.24	0.14	1.78 (1.66 to 1.91)	1.33 (1.23 to 1.43)
	Wave 1	560 (0.49)	1388 (0.27)	0.40	0.22	1.80 (1.63 to 1.98)	1.34 (1.21 to 1.49)
	Wave 2	455 (0.40)	1198 (0.24)	0.33	0.20	1.70 (1.53 to 1.90)	1.29 (1.15 to 1.44)
Death, COVID-19	Overall	377 (0.33)	815 (0.16)	0.08	0.04	2.07 (1.83 to 2.33)	1.23 (1.08 to 1.40)
	Wave 1	167 (0.15)	384 (0.08)	0.12	0.06	1.94 (1.61 to 2.32)	1.17 (0.97 to 1.42)
	Wave 2	193 (0.17)	404 (0.08)	0.14	0.07	2.14 (1.80 to 2.54)	1.26 (1.06 to 1.51)
RA							
Hospitalisation, COVID-19	Overall	693 (1.25)	1525 (0.66)	0.33	0.17	1.93 (1.77 to 2.11)	1.34 (1.22 to 1.48)
	Wave 1	361 (0.66)	779 (0.34)	0.54	0.28	1.96 (1.73 to 2.22)	1.36 (1.20 to 1.56)
	Wave 2	280 (0.52)	666 (0.29)	0.43	0.24	1.80 (1.56 to 2.06)	1.25 (1.08 to 1.45)
Death, COVID-19	Overall	297 (0.54)	564 (0.24)	0.14	0.06	2.23 (1.94 to 2.57)	1.30 (1.12 to 1.51)
	Wave 1	136 (0.25)	277 (0.12)	0.20	0.10	2.07 (1.69 to 2.54)	1.24 (1.00 to 1.54)
	Wave 2	147 (0.27)	272 (0.12)	0.23	0.10	2.30 (1.88 to 2.81)	1.32 (1.07 to 1.64)
Other IJD							
Hospitalisation, COVID-19	Overall	397 (0.66)	1212 (0.43)	0.17	0.11	1.53 (1.37 to 1.72)	1.29 (1.15 to 1.46)
	Wave 1	199 (0.33)	609 (0.22)	0.27	0.18	1.53 (1.30 to 1.79)	1.29 (1.09 to 1.52)
	Wave 2	175 (0.30)	532 (0.19)	0.24	0.16	1.54 (1.30 to 1.83)	1.33 (1.12 to 1.59)
Death, COVID-19	Overall	80 (0.13)	251 (0.09)	0.03	0.02	1.49 (1.16 to 1.92)	0.98 (0.76 to 1.27)
	Wave 1	31 (0.05)	107 (0.04)	0.04	0.03	1.35 (0.91 to 2.02)	0.89 (0.60 to 1.33)
	Wave 2	46 (0.08)	132 (0.05)	0.07	0.04	1.63 (1.16 to 2.28)	1.09 (0.77 to 1.53)

Note that results defined ‘overall’ follow patients from 1 January 2020 to 31 January 2021, and thus events cannot occur prior to wave 1. Wave1=1 March–30 June 2020, wave2=1 October 2020–31 January 2021.

HRs estimated from Cox proportional hazards models.

*Unadjusted accounts for age, sex and region via matching.

†Adjusted models are additionally adjusted for history of comorbidities (cancer, diabetes, heart failure, ischaemic heart disease, lung disease, kidney failure, stroke, surgery and venous thrombotic event), highest educational achievement, country of birth, marital status and number of hospitalisation days (previous year and previous 10 years).

IJD, inflammatory joint disease; RA, rheumatoid arthritis.

The absolute risks of the COVID-19-related outcomes with DMARDs were also similar across waves and treatment groups; no significant difference in HRs was detected. Descriptive statistics for treatment groups by wave are shown in online supplemental tables S6 and S7). For hospitalisation listing COVID-19 in IJD, the average risk (waves 1 and 2 combined) was 0.5% for the csDMARD group and 0.4% for the bDMARD/tsDMARD group (adjusted HR 0.89, 95% CI 0.68 to 1.17). Similarly, the average risk of death from COVID-19 was 0.2% for the csDMARD group and 0.1% for the bDMARD/tsDMARD group, adjusted HR 1.03, 95% CI 0.62 to 1.68; table 2). Regarding specific bDMARD/tsDMARDs, an increased HR for hospitalisation listing COVID-19 was detected for janus kinase inhibitors (JAKi) versus csDMARD (HR 1.99, 95% CI 1.18 to 3.35; table 2).

**Table 2:** Occurrence and relative risks of COVID-related events and other outcomes in individuals with chronic IJDs (rheumatoid arthritis, ankylosing spondylitis, psoriatic arthritis, other spondyloarthropathies and juvenile idiopathic arthritis) during wave 1 and wave 2 combined (March–June 2020 and October 2020–January 2021) according to DMARD treatment status at the beginning of each wave

		Events (N)	Crude risk (%), both waves	Crude risk (%), wave 1	Crude risk (%), wave 2	Adjusted HR*
IJD						
Hospitalisation, all causes	csDMARD	4212	5.9	6.1	5.8	Ref
	TNFi	1812	4.1	4.4	3.7	0.95 (0.87 to 1.03)
	Abatacept	187	7.0	6.3	7.5	0.92 (0.76 to 1.12)
	Tocilizumab	114	5.6	5.6	5.5	0.90 (0.71 to 1.15)
	Rituximab	362	8.5	9.1	7.7	1.21 (1.03 to 1.41)
	JAKi	224	6.3	6.3	6.2	0.92 (0.76 to 1.11)
	All b/tsDMARDs	2699	4.7	5.0	4.4	0.95 (0.88 to 1.02)
Hospitalisation, COVID-19	csDMARD	381	0.5	0.6	0.5	Ref
	TNFi	115	0.3	0.3	0.2	0.79 (0.58 to 1.08)
	Abatacept	9	0.3	0.4	0.3	0.58 (0.23 to 1.42)
	Tocilizumab	5	0.2	0.4	0.1	0.67 (0.22 to 2.08)
	Rituximab	42	1.0	1.0	1.0	1.43 (0.89 to 2.30)
	JAKi	31	0.8	1.0	0.7	1.99 (1.18 to 3.35)
	All b/tsDMARDs	202	0.4	0.4	0.3	0.89 (0.68 to 1.17)
Death, all-causes	csDMARD	588	0.8	0.9	0.8	Ref
	TNFi	104	0.2	0.2	0.2	0.60 (0.44 to 0.82)
	Abatacept	20	0.7	0.8	0.7	0.87 (0.49 to 1.54)
	Tocilizumab	10	0.5	0.6	0.4	0.72 (0.34 to 1.52)
	Rituximab	52	1.2	1.4	1.0	1.53 (1.00 to 2.35)
	JAKi	25	0.7	0.7	0.7	0.99 (0.56 to 1.73)
	All b/tsDMARDs	211	0.4	0.4	0.3	0.72 (0.55 to 0.94)
Death, COVID-19	csDMARD	127	0.2	0.2	0.2	Ref
	TNFi	19	0.04	0.03	0.1	0.81 (0.42 to 1.58)
	Abatacept	4	0.2	0.1	0.2	–
	Tocilizumab	2	0.1	0.2	0.0	–
	Rituximab	14	0.3	0.3	0.3	2.08 (0.94 to 4.60)
	JAKi	7	0.2	0.3	0.1	1.34 (0.49 to 3.65)
	All b/tsDMARDs	46	0.1	0.1	0.1	1.03 (0.62 to 1.68)
RA						
Hospitalisation, all-causes	csDMARD	3200	6.7	6.7	6.6	Ref
	TNFi	1048	5.0	5.3	4.6	0.89 (0.81 to 0.98)
	Abatacept	173	7.0	6.5	7.5	0.82 (0.67 to 1.00)
	Tocilizumab	107	5.7	5.9	5.5	0.82 (0.64 to 1.06)
	Rituximab	355	8.4	9.1	7.7	1.05 (0.89 to 1.23)
	JAKi	188	6.5	6.4	6.6	0.81 (0.66 to 0.99)
	All b/tsDMARDs	1871	5.8	6.0	5.5	0.88 (0.80 to 0.96)
Hospitalisation, COVID-19	csDMARD	294	0.6	0.6	0.6	Ref
	TNFi	62	0.3	0.4	0.2	0.75 (0.52 to 1.09)
	Abatacept	8	0.3	0.3	0.3	0.46 (0.18 to 1.21)
	Tocilizumab	5	0.3	0.4	0.1	0.75 (0.24 to 2.37)
	Rituximab	42	1.0	1.0	1.0	1.35 (0.83 to 2.22)
	JAKi	24	0.8	1.0	0.7	1.68 (0.92 to 3.05)
	All b/tsDMARDs	141	0.4	0.5	0.4	0.87 (0.64 to 1.20)
Death, all-causes	csDMARD	531	1.1	1.2	1.1	Ref
	TNFi	82	0.4	0.4	0.4	0.57 (0.41 to 0.79)
	Abatacept	19	0.8	0.9	0.6	0.69 (0.39 to 1.21)
	Tocilizumab	10	0.5	0.6	0.4	0.57 (0.27 to 1.18)
	Rituximab	51	1.2	1.4	1.0	1.04 (0.67 to 1.63)
	JAKi	23	0.8	0.7	0.9	0.80 (0.44 to 1.45)
	All b/tsDMARDs	185	0.6	0.6	0.5	0.64 (0.48 to 0.86)
Death, COVID-19	csDMARD	117	0.2	0.2	0.3	Ref
	TNFi	15	0.07	0.1	0.1	0.71 (0.35 to 1.42)
	Abatacept	4	0.2	0.1	0.2	–
	Tocilizumab	2	0.1	0.2	0	–
	Rituximab	14	0.3	0.3	0.3	1.46 (0.68 to 3.14)
	JAKi	7	0.2	0.4	0.1	1.09 (0.40 to 2.95)
	All bDMARD/tsDMARDs	42	0.1	0.1	0.1	0.88 (0.52 to 1.47)

*Adjusted HRs were estimated from inverse probability of treatment-weighted Cox regression models where weights accounted for history of comorbidities (cancer, diabetes, heart failure, ischaemic heart disease, lung disease, kidney failure, stroke, surgery and venous thrombotic event), highest educational achievement, country of birth, marital status, number of hospitalisation days (previous year and previous 10 years), additional adjustment of previous bDMARD/tsDMARD use, number of previous bDMARD/tsDMARDs, concomitant use of csDMARDs and steroids were included in the Cox regression. Note that HRs are not presented where events are <5.

bDMARD, biological disease-modifying antirheumatic drug; csDMARD, conventional synthetic disease-modifying antirheumatic drug; DMARD, disease-modifying antirheumatic drug; Ref, reference; TNFi, Tumor Necrosis Factor inhibitors; tsDMARD, targeted synthetic disease-modifying antirheumatic drug.

### Part 2: effects of the COVID-19 pandemic on care provision for IJDs

#### Observed incidence of IJDs

**Figure 2:**
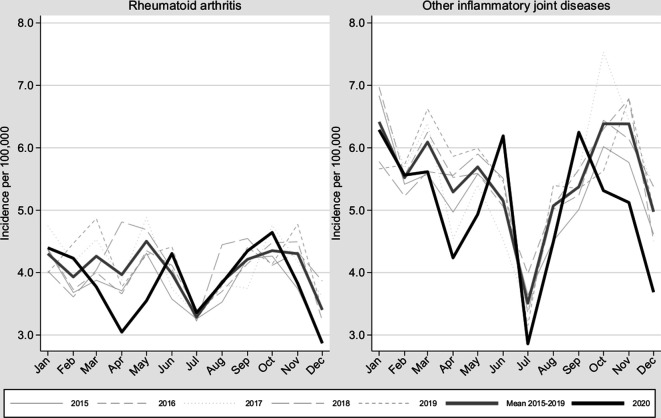
Incidence of rheumatoid arthritis and of the combined group of all inflammatory joint diseases in Sweden during 2020 vs 2015–2019.

Figure 2 displays the monthly incidence of RA and of all IJD for 2015–2020. The pattern during 2020 was largely similar to those of previous years (a dip coinciding with Swedish holiday periods) but also included a visible dip during March–May 2020 and a possible dip coinciding with the second wave. Online supplemental figure S1 displays the same data but longitudinally. Comparing the average of 2020 to the average across 2015–2019, we found that there was a 7% decrease in IJD incidence, but this decrease was part of a downward trend over several years.

#### Visits in specialised care

**Figure 3:**
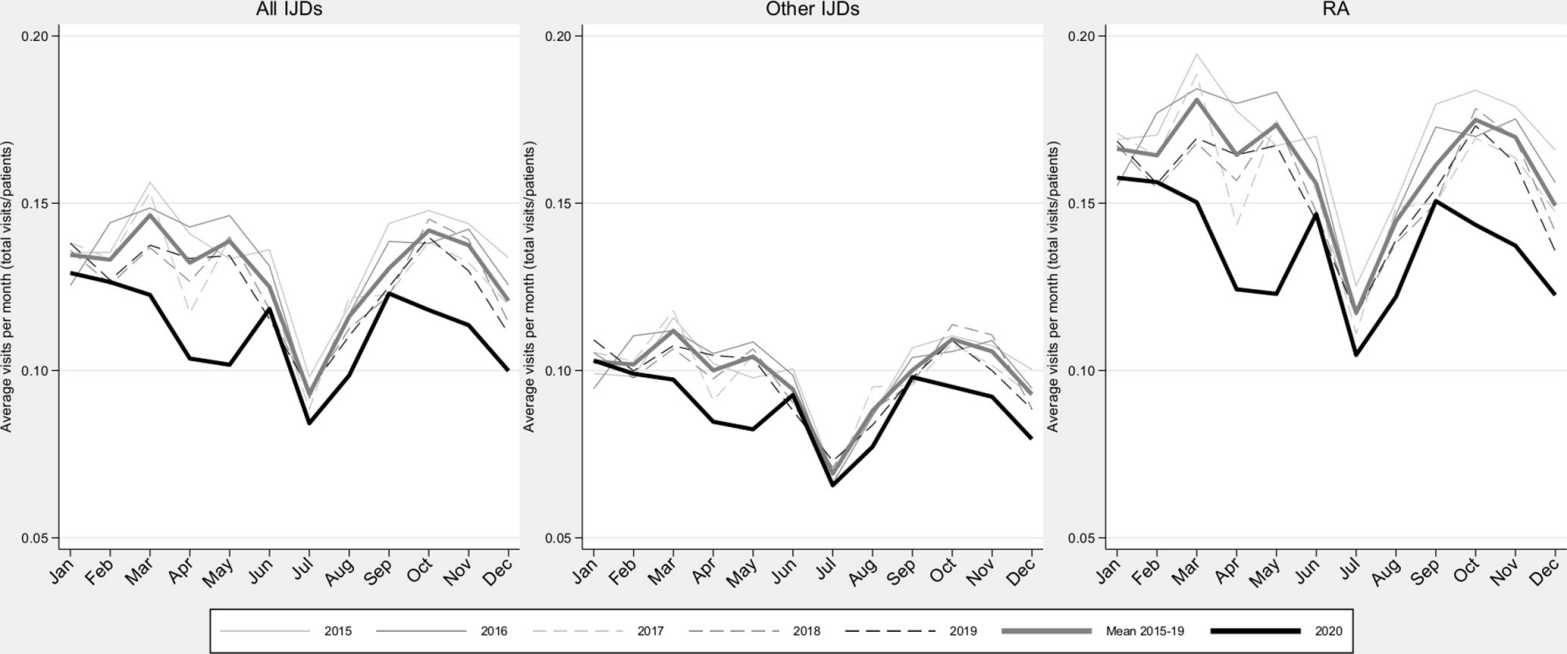
Average number of visits to rheumatology or internal medicine per month (total number of visits/patients alive at the beginning of each month) for patients with RA and other IJDs in Sweden between 2015 and 2020. IJD, inflammatory joint disease; RA, rheumatoid arthritis.

Figure 3 presents the average number of rheumatologist visits among patients with IJD for 2015–2020. The average visit rate in 2020 was 16% lower than the average for 2015–2019, but the annual average visit rate decreased each year 2015–2019, with 2020 representing an exaggeration of this decreasing trend, in particular during waves 1 and 2. It should be noted that in parallel to this reduction, there was a significant shift in the proportion of physical versus distance visits from around 25% distance visits prepandemic, to a peak at just above half of all visits during the pandemic (T Frisell, personal communication at the National Board of Health and Welfare, 2021). Online supplemental figure S2 displays the same data as Figure 3 but expresses the visits during 2020 as proportions of ‘expected’ visits based on data from 2015 to 2019. Similar patterns of reduction in average number of visits were seen when concentrating on visits listing an ICD-10 code for IJD (online supplemental figure S3). Exploratory analyses revealed a larger proportional decline in visit intensity versus the average 2015–2019 in early versus established IJD and among patients above 70 years (online supplemental figure S4 and S5).

#### Use of DMARDs

**Figure 4:**
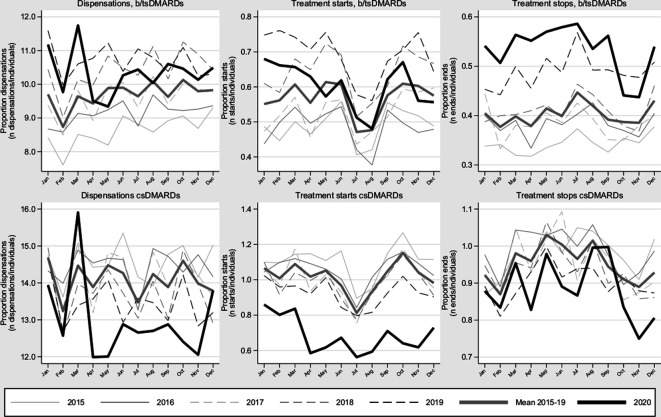
Dispensations, treatment starts and treatment stops of bDMARDs/tsDMARDs and csDMARDs across 2015–2020 for all inflammatory joint diseases in Sweden. presented monthly, as a proportion of all patients at risk per month. bDMARDs/tsDMARDs: adalimumab, certolizumab pegol, golimumab, etanercept, infliximab, abatacept, anakinra, sarilumab, tocilizumab, rituximab, tofacitinib, baricitinib, upadacitinib and apremilast. csDMARDs: sulfasalazine, methotrexate, hydroxychloroquine and leflunomide. bDMARD, biological disease-modifying antirheumatic drug; csDMARD, conventional synthetic disease-modifying antirheumatic drug; tsDMARD, targeted synthetic disease-modifying antirheumatic drug.

Figure 4 presents dispensations, treatment starts and treatment stops for bDMARD/tsDMARDs and csDMARDs annually for 2015–2020 (see online supplemental table S8 for specific DMARDs). For dispensations and treatment starts, there was an increasing trend of DMARD use for 2015–2019 that did not continue during 2020. Following an initial peak in dispensations around the beginning of the first wave, bDMARD/tsDMARD dispensations and treatment starts were both lower than during 2019. Due to the underlying trend, dispensations during 2020 were still 6.5% higher compared with the average 2015–2019. For bDMARD/tsDMARD discontinuations, the increasing trend from previous years was accentuated during 2020. For csDMARDs, we noted a slight trend towards reduced dispensations that was accentuated during 2020 (−8.5%), a marked drop in treatment starts and an accompanying but less pronounced drop in treatment discontinuation.

#### Observed incidence of comorbidities

**Figure 5:**
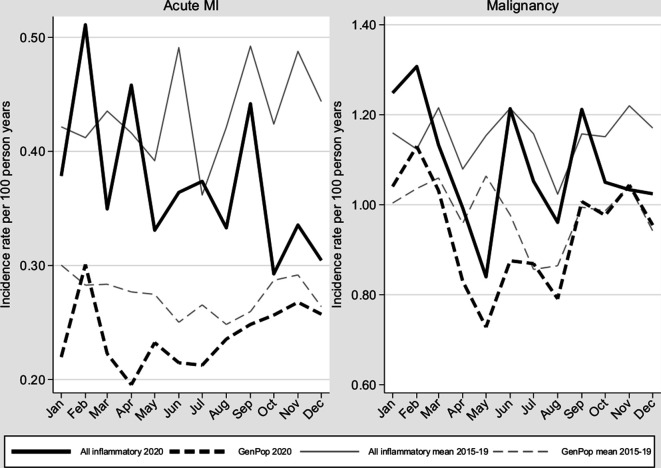
Incidence of acute MI and malignancies in patients with all inflammatory joint diseases and matched general population comparators in Sweden, during 2020 vs 2015–2019. MI, myocardial infarction.

Figure 5 presents the incidence of first acute MI and of first diagnosis of any cancer among patients with IJD and population comparators for 2020 and across 2015–2019. Both outcomes saw a dip during the first wave for comparators and IJD, with moderate variability in the latter. Note that due to the first event per year definition, a decrease over each year is expected, but comparisons over years are possible. Overall, 2020 saw a 15% decrease in MI diagnoses versus the average 2015–2019 for both IJD and comparators; a 5% decrease was seen for cancer diagnoses.

## Discussion

Our results on COVID-19 outcomes indicate a consistency in both absolute and relative risks of hospitalisation and death following COVID-19 between the first and the second waves, and a general absence of strong effects of treatment with bDMARDs/tsDMARDs, with the possible exception of JAKi and rituximab. Our results on effects of the COVID-19 pandemic on care provision demonstrated (1) only a minor decline in the incidence of new-onset IJD during 2020, which rather appears to reflect an underlying secular trend than a distinct effect of the pandemic itself; (2) a less than 20% decline in the overall number of visits among patients with IJD during 2020; (3) marked deviations during 2020 from strong underlying trends towards more use and higher turnover of bDMARDs/tsDMARDs (and lower use of csDMARDs); and (4) a similar drop in the incidence of first cancer and MI in IJD as in the general population.

Regarding severe COVID-19 outcomes, temporal trends were found in studies with focus on the first wave of the pandemic for comparisons within patients with IJD^14 15^; we did not find such patterns when comparing patients with IJD to population comparators between the two waves. Previous studies have reported signals of increased relative risks for adverse outcomes for patients treated with JAKi or rituximab.^16^ Our current results are in line with these, and extend these by providing measures on absolute risks for each outcome and drug.

In contrast to the increasing evidence regarding outcomes of SARS-CoV-2 infections in individuals with IJD and with DMARDs, much less is known respecting the effects of the pandemic on the care for patients with incident or prevalent IJD. We are not aware of previous studies assessing the observed incidence of IJD during the pandemic. Our study suggests only a modest decline that largely seems to be driven by factors other than the pandemic.

In light of the early notion that both IJD and DMARDs might aggravate SARS-CoV-2 infection, patients with IJD may have been particularly reluctant to (physically) visit healthcare. A US study reported that 57% of patients with rheumatic diseases avoided in-person visits during March–May 2020,^17^ and large reductions in physical consultations were observed in Danish patients with inflammatory arthritis.^18^ The decline in the rate of registered visits in our study was more modest, which, besides country-specific differences, may reflect that we assessed rates throughout 2020 rather than selectively during the height of the (first) wave. Further, a part of the observed decline during 2020 was expected based on trends from preceding years.

Reductions in DMARD use have been reported in other studies.^17 19–22^ Our results regarding 2020 suggest marked changes compared with 2019 (decreasing starts and increasing stops of bDMARDs/tsDMARDs); such changes in dispensations could be affected by treatment hoarding or prioritisation of use for COVID-19. However, the observed changes in dispensations were not only related to the COVID-19 pandemic but also in reality even more pronounced as they also contained a deviation from underlying secular trends in the opposite direction. These observations underscore the need to put data from the pandemic into a longitudinal perspective.

We are not aware of previous studies that have assessed the diagnostic intensity regarding comorbidities. Our results for the general population verified declines during Spring 2020 reported elsewhere^9 10 23^ and suggest a similar pattern in IJD.

We could assess COVID-19-related outcomes but not risks for SARS-CoV-2 infection. We cannot formally rule out that some of the observed effects would stem from an increased risk of primary infection. We used both comorbidity and other data to accommodate confounding by indication, yet the increased risks observed for JAKi and signals observed for rituximab may contain residual confounding. We could capture visits to healthcare but could not determine if, and which, visits were telephone and/or internet consultations, nor the content of any communication. However, the total number of visits recorded in the NPR was similar in 2020 compared with 2019 (T Frisell, personal communication at the National Board of Health and Welfare, 2021), indicating that coverage of patient visits should not majorly affect our results. Regarding DMARDs, we used dispensations rather than prescriptions but could not verify actual drug use. Thus, our results regarding DMARDs may be conservative. From an international perspective, the cumulative incidence of, and death from, COVID-19 in Sweden has been comparable to many other countries. Our results on COVID-19 outcomes should be generalisable to many other countries with a similar epidemic ‘pressure’. Since different countries have instituted different public restrictions at different times, our results regarding the impact on care provision, and similarities between waves, may not be universally applicable.

Our study population effectively comprised all patients with IJD in Sweden and matched comparators, all of whom could be followed up longitudinally using prospectively recorded data. In contrast to many previous studies on COVID-19 in IJD, we did not have to rely on self-reported data but could use external and virtually complete data sources. We could also assess effects not only during the first wave but also throughout 2020. This allowed for estimation of both relative and absolute risks, for comparisons of the two waves to each other, of IJD to the general population and of 2020 not only to the previous year but also in relation to underlying secular trends over the preceding years. With regard to effects of the pandemic on rheumatology care provision, we could study several important aspects (diagnosis of IJD, health encounters among those with IJD, DMARD use and diagnosis of comorbidities).

To conclude, the absolute and relative risks of hospitalisation or death from COVID-19 in patients with IJD compared with the general population were equally elevated during the second wave as during the first wave of the COVID-19 pandemic. Our results reinforce that the increased risk of hospitalisation and death, at least in part, may be explained by other (comorbid) conditions than the IJD per se, and rather than bDMARDs/tsDMARDs use. In terms of effects of the pandemic on care provision for IJD, some of the observed effects (incidence of IJD, decreased rate of rheumatologist visits) were of modest size and part of underlying secular trends over several years rather than distinct effects of the pandemic. Others (increasing use of bDMARDs/tsDMARDs and decreasing use of csDMARD in 2020) were more marked but must again be interpreted in light of strong secular trends.

## Data Availability

No data are available.

## References

[1] Bower H , Frisell T , Di Giuseppe D , et al . Impact of the COVID-19 pandemic on morbidity and mortality in patients with inflammatory joint diseases and in the general population: a nationwide Swedish cohort study. Ann Rheum Dis 2021:annrheumdis-2021-219845. 10.1136/annrheumdis-2021-219845 PMC8206171 33622688

[2] D'Silva KM , Jorge A , Cohen A , et al . COVID-19 outcomes in patients with systemic autoimmune rheumatic diseases compared to the general population: a US multicenter, comparative cohort study. Arthritis Rheumatol 2021;73:914–20. 10.1002/art.41619 33305544 PMC8169514

[3] Gianfrancesco M , Yazdany J , Robinson PC . Epidemiology and outcomes of novel coronavirus 2019 in patients with immune-mediated inflammatory diseases. Curr Opin Rheumatol 2020;32:434–40. 10.1097/BOR.0000000000000725 32675715

[4] England BR , Roul P , Yang Y , et al . Risk of COVID-19 in rheumatoid arthritis: a national Veterans Affairs matched cohort study in at-risk individuals. Arthritis Rheumatol 2021. 10.1002/art.41800. [Epub ahead of print: 05 May 2021]. PMC8239709 33955209

[5] Care and covid-19: mortality among inpatient care (Vård och covid-19: Dödlighet Bland slutenvårdade): the public health agency (Socialstyrelsen). Available: https://www.socialstyrelsen.se/statistik-och-data/statistik/statistik-om-covid-19/statistik-om-slutenvard-av-patienter-med-covid-19/ [Accessed 19 July 2021].

[6] Gianfrancesco M , Hyrich KL , Al-Adely S , et al . Characteristics associated with hospitalisation for COVID-19 in people with rheumatic disease: data from the COVID-19 global rheumatology alliance physician-reported registry. Ann Rheum Dis 2020;79:859–66. 10.1136/annrheumdis-2020-217871 32471903 PMC7299648

[7] Putman M , Chock YPE , Tam H , et al . Antirheumatic disease therapies for the treatment of COVID-19: a systematic review and meta-analysis. Arthritis Rheumatol 2021;73:36–47. 10.1002/art.41469 32741139 PMC7435536

[8] Socialstyrelsen (The National Board of Health and Welfare) . Analysis of unmet care needs after the pandemic. Available: https://www.socialstyrelsen.se/statistik-och-data/statistik/pandemins-effekter-pa-varden/analys-uppdamda-vardbehov-efter-pandemin/ [Accessed 19 Aug 2021].

[9] Eijkelboom AH , de Munck L , Vrancken Peeters M-JTFD , et al . Impact of the COVID-19 pandemic on diagnosis, stage, and initial treatment of breast cancer in the Netherlands: a population-based study. J Hematol Oncol 2021;14:64. 10.1186/s13045-021-01073-7 33865430 PMC8052935

[10] Mohammad MA , Koul S , Olivecrona GK , et al . Incidence and outcome of myocardial infarction treated with percutaneous coronary intervention during COVID-19 pandemic. Heart 2020;106:1812–8. 10.1136/heartjnl-2020-317685 33023905 PMC7677488

[11] Weekly surveillance summary . Week 27, 2021. COVID-19 country overviews.: World health organisation (who). Available: https://covid19-country-overviews.ecdc.europa.eu/#35_Sweden [Accessed 19 Jul 2021].

[12] van Vollenhoven RF , Askling J . Rheumatoid arthritis registries in Sweden. Clin Exp Rheumatol 2005;23:S195–200. 16273807

[13] Sweden S . Statistikmyndigheten SCB). Statistical Database. Population per month by region, age and sex.

[14] Jorge A , D'Silva KM , Cohen A , et al . Temporal trends in severe COVID-19 outcomes in patients with rheumatic disease: a cohort study. Lancet Rheumatol 2021;3:e131–7. 10.1016/S2665-9913(20)30422-7 33392516 PMC7758725

[15] Serling-Boyd N , D'Silva KM , Hsu TY , et al . Coronavirus disease 2019 outcomes among patients with rheumatic diseases 6 months into the pandemic. Ann Rheum Dis 2021;80:660–6. 10.1136/annrheumdis-2020-219279 33257496 PMC7705424

[16] Sparks JA , Wallace ZS , Seet AM , et al . Associations of baseline use of biologic or targeted synthetic DMARDs with COVID-19 severity in rheumatoid arthritis: results from the COVID-19 global rheumatology alliance physician registry. Ann Rheum Dis 2021;80:1137–46. 10.1136/annrheumdis-2021-220418 34049860 PMC8172266

[17] George MD , Venkatachalam S , Banerjee S , et al . Concerns, healthcare use, and treatment interruptions in patients with common autoimmune rheumatic diseases during the COVID-19 pandemic. J Rheumatol 2021;48:603–7. 10.3899/jrheum.201017 33191284 PMC8121899

[18] Glintborg B , Jensen DV , Terslev L , et al . Impact of the COVID-19 pandemic on treat-to-target strategies and physical consultations in &gt;7000 patients with inflammatory arthritis. Rheumatology 2021;60:SI3–12. 10.1093/rheumatology/keab500 34146099 PMC8344418

[19] Mancuso CA , Duculan R , Jannat-Khah D , et al . Modifications in systemic rheumatic disease medications: patients' perspectives during the height of the COVID-19 pandemic in New York City. Arthritis Care Res 2021;73:909–17. 10.1002/acr.24489 33085850

[20] Hausmann JS , Kennedy K , Simard JF , et al . Immediate effect of the COVID-19 pandemic on patient health, health-care use, and behaviours: results from an international survey of people with rheumatic diseases. The Lancet Rheumatology 2021;3:e707–14. 10.1016/S2665-9913(21)00175-2 34316727 PMC8298011

[21] Belleudi V , Rosa AC , Poggi FR , et al . Direct and indirect impact of COVID-19 for patients with immune-mediated inflammatory diseases: a retrospective cohort study. J Clin Med 2021;10. 10.3390/jcm10112388. [Epub ahead of print: 28 05 2021]. PMC8197915 34071452

[22] George MD , Baker JF , Banerjee S , et al . Social distancing, health care disruptions, telemedicine use, and treatment interruption during the COVID-19 pandemic in patients with or without autoimmune rheumatic disease. ACR Open Rheumatol 2021;3:381–9. 10.1002/acr2.11239 33934576 PMC8207682

[23] Fallara G , Sandin F , Styrke J , et al . Prostate cancer diagnosis, staging, and treatment in Sweden during the first phase of the COVID-19 pandemic. Scand J Urol 2021;55:184–91. 10.1080/21681805.2021.1910341 33913376

