# Supplementary material for: Effects of the COVID-19 pandemic on patients with inflammatory joint diseases in Sweden: from infection severity to impact on care provision
Source: RMD Open. 2021 Dec 7;7(3):e001987. doi: 10.1136/rmdopen-2021-001987 (PMC8655349; doi:10.1136/rmdopen-2021-001987)

Supplementary Materials

Supplementary Methods

Propensity scores were calculated for use as weights within the inverse probability weighted Cox proportional hazards models[1]. These propensity scores were estimated separately for treatment cohorts for each wave within the 1) all inflammatory joint diseases, and 2) RA cohorts. Propensity scores were also calculated separately when comparing the csDMARD to the b/tsDMARD groups in each of the three inflammatory joint disease cohorts.

Multinomial logistic (for the estimation of propensity scores within the six DMARD cohorts), and logistic regression models (for the estimation of propensity score within the csDMARD/b-tsDMARD cohorts) were fitted. All models contained the same covariates: history of cancer, diabetes, heart failure, ischemic heart disease, hospitalization listing infection, lung disease, stroke, venous thromboembolic events, kidney failure, and surgery, age, sex, disease duration, DAS28, days in hospital (both in the previous 10 years, and 1 year), region of domicile, educational level, civil status, and country of birth. See Supplementary Table S4 for definitions and the functional form of each of the covariates included in the propensity score model.

Stabilised inverse probability of treatment weights[2] were calculated from the propensity scores predicted from these models and were were additionally restricted to be no larger than the 99% and no smaller than the 1% centile of the distribution to further avoid extreme weight. The standardised mean bias[3] was used to determine whether balance had been reached when using stabilized weights. Subsequent Cox proportional hazards models used the robust sandwich estimator to calculate standard errors; these models were additionally adjusted for an indicator variable specifying whether the individual had received a different b/tsDMARD in the 180 days before start of follow-up, the number of previous b/tsDMARDs, and concomitant csDMARD and steroid use.

[1]Austin PC. The performance of different propensity score methods for estimating marginal hazard ratios. Stat. Med 2012:32(12) 2837-2849. doi: 10.1002/sim.5705
[2]Pezzi A, Cavo M, Biggeri A et al. Inverse probability weighting to estimate causal effect of a singular phase in a multiphase randomized clinical trial for multiple myeloma. BMC Medical Research Methodology 2016:16(150). doi: 10.1186/.s12874-016-0253-9
[3]Zhang Z, Kim HJ, Lonjon G et al. Balance diagnostics after propensity score matching. Ann Transl Med 2019:(1)16. doi:10.21037/atm.2018.12.10

Table S1. Data sources included in the study

**Swedish Rheumatology Quality Register (SRQ)**	A nationwide longitudinal clinically integrated register operated by The Swedish Society for Rheumatology, started in 1996. Patients with RA and other rheumatologic diseases are registered in the SRQ by the treating rheumatologist. SRQ contains information about disease activity and additional information such as treatment and smoking status. SRQ covers 95% of all patients with RA treated with b/tsDMARDs in Sweden.
**Swedish Patient Register (NPR)**	A national register maintained by The National Board of Health and Welfare. Hospital discharges from inpatient care and patients visits in non-primary outpatient care, have been registered, since 1964 and 2001 respectively. Diagnoses are coded according to the Swedish version of the International Classification of Disease (ICD). The coverage of the inpatient part is close to 100%, for the outpatient part, the overall coverage is around 80% (higher for public than for private care-providers).
**Prescribed Drug Register (PDR)**	A national register maintained by The National Board of Health and Welfare. It contains information about all drugs dispensed on prescription in Sweden and is linked to the personal identification number since 2005. The coverage is close to 100%.
**Swedish Population Register**	A national register maintained by Swedish Tax agency. Contains information such as home district, civil status and migration data.
**Longitudinal database for insurance and labor market-studies (LISA)**	A national register maintained by Statistics Sweden. It contains information about sick leave, parental leave and employment status in Sweden from 1990.
**Cause of Death Register**	The Cause of Death Register is a national register containing information on date and cause of death (underlying and contributory) for all deceased residents, including deaths among Swedish residents who died abroad. The register was started in 1952, and the data is considered complete since 1961. From that year and onward, cause of death is missing for less than 0.5% of deceased individuals, and in 2002, a validation study estimated that only 3.3% had any errors at the three-digit level of the ICD-coded underlying cause of death.

Table S2: ICD10 and ATC codes used to define cohorts

**Inflammatory joint disease cohort definitions**		**DMARD treatment cohort definitions***	
**Disease**	**ICD10 code**	**DMARD**	**ATC code**
Rheumatoid arthritis	M05, M06	csDMARD	L04AX01, A07EC01, L04AD01, P01BA01, M01CB01, L04AA06, L04AX03, L01AA01, P01BA02, J01AA08, L04AA13, M01CC01
Psoriatic arthritis	M070, M071, M073, L405	TNFi	L04AB04, L04AB05, L04AB01, L04AB06, L04AB02
Ankylosing spondylitis	M45	Abatacept	L04AA24
Other spondyloarthropathies	M460, M461, M468, M469	Tocilizumab	L04AC07
Juvenile idiopathic arthritis	M08, M09	Rituximab	L01XC02
		JAKi	L04AA29, L04AA37, L04AA44

*As recorded in the Prescribed Drug Register and Swedish Rheumatology Quality Register

Table S3: Outcome definitions

**Outcome**	**ICD10 code**	**Data source**
Effects of COVID-19 infection		
Hospitalization, COVID-19	U071, U072	National Patient Register, inpatient component. Both main and secondary diagnoses codes.
Death, COVID-19	U071, U072	Swedish Cause of Death Register. Both main and contributory causes of death codes.
Diagnoses of co-morbid conditions in patients with inflammatory joint diseases		
Acute myocardial infarction (MI)	121 122	National Patient Register, inpatient and outpatient components. Main diagnosis only. (Note in analysis the first MI per individual per year was included, with no MI diagnosis in the previous 10 years)
Malignancy	C00-C97 (minus C44)	National Patient Register, inpatient and outpatient components. Main diagnosis only. (Note in analysis the first cancer per individual per year was included, with no cancer diagnosis in the previous 10 years)
Use of DMARDs (dispensation, start, stop)		
**b/tsDMARDs**		Prescribed drug register using dispensations of the specific ATC codes. Dispensations ends were defined as the defined daily dose per package, multiplied by the total number of packages plus 90 days. Treatment episodes were calculated using the dispensations and dispensation ends, where a new episode was defined at more than 93 days gap between end and dispensation, or a start of another b/tsDMARD.
**TNFi**		
Adalimumab	L04AB04	
Certolizumab pegol	L04AB05	
Golimumab	L04AB06	
Etanercept	L04AB01	
**Abatacept**	L04AA24	
**IL-inhibitors**		
Anakinra	L04AC03	
Sarilumab	L04AC14	
Tocilizumab	L04AC07	
**JAKi**		
Tofacitinib	L04AA29	
Baricitinib	L04AA37	
Upadacitinib	L04AA44	
Apremilast	L04AA32	
**csDMARDs**		

Sulfasalazine	A07EC01	Prescribed Drug Register. As above, but treatment episodes did not end at a start of another treatment.
Methotrexate	L04AX03	
Hydroxychloroquine	P01BA02	
Leflunomide	L04AA13	
**Other**		
Prednisolone	H02AB06	
Guselkumab	L04AC16	
Secukinumab	L04AC10	
Ixekizumab	L04AC13	

Table S4: Description of covariates included in the propensity score estimation model.

Variable	Description
**Comorbidity**	
History of cancer	History of cancer recorded within 5 years prior to cohort entry. Data retrieved from the Cancer Register.<br>Indicator variable (Y/N). Note that information on cancer diagnoses recorded in the Swedish Cancer Register was only available until December 31st 2018.
History of diabetes	History of diabetes recorded in the 10 years recorded prior to cohort entry. Defined as a record in the National Patient Register (inpatient and outpatient components, ICD10: E10-E11) or dispensation of treatment (ATC: A10) in the Prescribed Drug Register.<br>Indicator variable (Y/N).
History of heart failure	History of heart failure recorded in the 5 years recorded prior to cohort entry. Defined as record in National Patient Register (inpatient component, ICD10: I50).<br>Indicator variable (Y/N).
History of ischemic heart disease.	History of ischemic heart disease recorded in the 5 years recorded prior to cohort entry. Defined as record in National Patient Register (inpatient component, ICD10: I20-I25).<br>Indicator variable (Y/N).
History of hospitalized infections	History of infections recorded in the 2 years prior to cohort entry. Defined as recorded in National Patient Register (inpatient component, ICD10: A00-B99, D73.3, E06.0, E32.1, G00-G02, G04.2, G05-G07, H00.0, H44.0, H60.0-H60.3, H66-H67, H70, I30.1, I40.0, J00-J22, J32, J34.0, J36, J38.3, J39.0-J39.1, J44.0, J85, J86, K04.4, K04.6, K04.7, K10.2, K11.3, K12.2, K14.0, K57.0, K57.2, K57.4, K57.8, K61, K63.0, K65.0, K65.1, K65.2, K65.9, L00-L08, L30.3, M00-M01, M46.2-M46.5, M60.0, M65.0, M71.0, M71.1, M72.6, M86, N10, N11, N12, N13.6, N15.1, N15.9, N30.0 N30.8, N34.0, N41.2, N43.1, N45.2, N45.3, N45.4, N48.2, N61, N70, N73, N75.1).<br>Indicator variable (Y/N).
History of lung disease	History of lung disease other than infectious pneumonia recorded in the 5 years recorded prior to cohort entry. Defined as record in National Patient Register (inpatient and outpatient components, ICD10: J40-J94).<br>Indicator variable (Y/N).
History of kidney failure	History of kidney failure recorded in the 5 years recorded prior to cohort entry. Defined as record in National Patient Register (inpatient and outpatient components, ICD10:N17-N19).<br>Indicator variable (Y/N).
History of stroke	History of stroke recorded in the 5 years recorded prior to cohort entry. Defined as record in National Patient Register (inpatient and outpatient components, ICD10:I50-I69).<br>Indicator variable (Y/N).

History of joint surgery	History of joint surgery recorded in the 10 years prior to cohort entry. Defined as record in National Patient Register (inpatient and outpatient components, operational codes: NGB, NFB, NBB, NHB, NHC, NHE, NHF, NHG, 8423, 8424, 8426, 8419, 8437, 8436, 8420, 8421, 8422, 8400-8415).<br>Indicator variable (Y/N).
History of venous thrombotic event	History of VTE recorded in the 5 years recorded prior to cohort entry. Defined as record in National Patient Register (inpatient component, ICD10:I82, I26).<br>Indicator variable (Y/N).
**Health-care resource utilization**	
Hospital days in the previous year	The number of days spent in hospital during the 365 days prior to cohort entry. Data obtained from the inpatient component of the National Patient Register.<br>Categorized into 0, 1-3, and 4+ days.
Hospital days in the previous 10 years	The number of days spent in hospital during the period 10 years to 365 days prior to cohort entry. Data obtained from the inpatient component of the National Patient Register. Categorized into 0, 1-6, and 7+ days.
**Socioeconomics**	
Education	Highest education achieved as recorded in the year prior to cohort entry. Data obtained from the Longitudinal integrated database for health insurance and labour market studies (LISA). Note that education information was only available to 2018 in LISA so the value in 2018 was assumed for any subsequent years. Categorized into:<br>1= <9 years<br>2=9-12years<br>3=12years+
Civil status	Civil status recorded in the year prior to cohort entry. Data obtained from LISA, Note that civil status information was only available to 2017 in LISA so the value in 2017 was assumed for any subsequent years. Categorized into married/partner, or single.
Country of birth	Country of birth obtained from the Total Population Register categorized as Sweden, rest of Europe, and rest of world.
**Disease-related**	
DAS28	DAS28 value (ESR) from most recent rheumatology visit recorded in the SRQ within one year prior to start of follow-up.<br>Categorized into remission (<2.6), low (2.6-3.1), moderate (3.2- 5.1), high (5.2+), and missing.
Disease duration	Disease duration in years, taken as the difference between the diagnosis date (defined using the disease selection definition and data in the National Patient register) and entry to cohort.<br>Categorized as <2, 2-4, 5-9, 10+ years.
**Treatment-related**	

Number of previous b/tsDMARDs*	Number of previous b/tsDMARDs prior to the treatment that caused entry to cohort. Identified by combining the PDR and SRQ. Categorized as 0, 1-2, 3+.
b/tsDMARD recorded in the previous 180 days*	Identifies if a different b/tsDMARD was recorded in the previous 180 days prior to start.<br>Indicator variable (Y/N).
Concomitant steroid use*	Dispensation of steroids (ATC: H02AB06) recorded in the Prescribed Drug Register in the 90 days prior to cohort entry.
Concomitant csDMARD use*	Concomitant csDMARD use defined as dispensation of csDMARD recorded in the Prescribed Drug Register within the 120 days prior to cohort entry where the dispensation occurs after the order date of the treatment defining the exposure cohort (ATC codes: L04AX01, A07EC01, L04AD01, P01BA01, M01CB01, L04AA06, L04AX03, L01AA01, P01BA02, J01AA08, L04AA13, M01CC01)

*Variables included in weighted Cox model not propensity score estimation model

Table S5: Descriptive statistics of inflammatory joint disease patients by treatment cohort in Sweden during the first wave of the COVID-19 pandemic (March 2020- June 2020)

	csDMARD	TNFi	Abatacept	Tocilizumab	Rituximab	JAKi	All b/tsDMARDs	General population
N individuals	34725	22530	1323	1037	2177	1740	28807	274875
Age at entry, median (IQR)	67 (55-75)	54 (42-66)	65 (54-73)	62 (50-72)	68 (58-75)	60 (49-69)	56 (44-68)	61 (48-71)
Female	64%	58%	79%	79%	77%	79%	62%	63%
Disease duration, median years (IQR)	8.5 (3.9-14.4)	9.4 (4.5-15.3)	12.1 (6.4-17.6)	11.8 (6.8-17.4)	13.6 (8.8-18.2)	11.0 (5.6-16.9)	10.1 (5.0-16.0)	N/A
DAS28, median (IQR)	2.6 (1.9-3.4)	2.4 (1.7-3.4)	3.0 (2.4-4.3)	1.9 (1.1-3.2)	2.9 (2.1-3.9)	3.3 (2.4-4.4)	2.5 (1.8-3.6)	N/A
Missing DAS28	73%	63%	55%	58%	49%	52%	61%	N/A
Number of previous b/tsDMARDs, median (IQR)	0 (0-0)	0 (0-0)	0 (0-1)	0 (0-1)	0 (0-1)	0 (0-1)	0 (0-0)	N/A
Other b/tsDMARD use in 180 days prior to entry	0%	2%	9%	10%	5%	6%	3%	
Concomitant csDMARD use	100%	37%	42%	30%	37%	31%	37%	1%
Concomitant steroid use	22%	15%	37%	33%	31%	34%	19%	2%
History of comorbidity*								
Cancer	6%	2%	3%	2%	6%	3%	2%	4%
Diabetes	14%	9%	15%	11%	15%	12%	10%	10%
Heart failure	3%	1%	4%	2%	4%	2%	2%	2%
Ischemic heart disease	6%	3%	7%	3%	7%	5%	4%	3%
Infection	6%	3%	9%	5%	10%	8%	4%	2%
Lung disease	9%	6%	17%	10%	18%	11%	8%	5%
Kidney failure	3%	2%	3%	3%	3%	2%	2%	1%
Stroke	3%	2%	3%	2%	3%	2%	2%	2%
Surgery	14%	12%	25%	24%	26%	22%	15%	6%
VTE	1%	1%	1%	1%	2%	1%	1%	1%
Highest education achieved								

<9 years	14%	5%	11%	8%	11%	7%	6%	9%
10-12 years	58%	58%	57%	59%	59%	59%	58%	55%
12+ years	28%	37%	32%	34%	29%	34%	36%	36%
Missing	1%	0%	1%	0%	1%	0%	0%	1%
Married/partner (%)	51%	49%	52%	49%	49%	49%	49%	48%
Born in Sweden (%)	90%	89%	90%	90%	86%	89%	89%	87%
Missing country of birth (%)	2%	2%	2%	2%	2%	2%	2%	3%

*See Supplementary Table S4 for definitions
csDMARD= conventional synthetic disease modifying anti-rheumatic drug, b/tsDMARD= biological/targeted disease modifying anti-rheumatic drug, DAS28=disease activity score on 28 joints, JAKi= Janus kinase inhibitors, N/A=not applicable, TNFi= tumor necrosis factor inhibitor, VTE=veneous thromboetic event

Table S6: Descriptive statistics of inflammatory joint disease patients by treatment cohort in Sweden during the second wave of the COVID-19 pandemic (October 2020- January 2021)

	**csDMARD**	**TNFi**	**Abatacept**	**Tocilizumab**	**Rituximab**	**JAKi**	**All b/tsDMARDs**	**General population**
N individuals	36366	22249	1366	1016	2105	1836	28572	280462
Age at entry, median (IQR)	67 (55-76)	55 (43-66)	65 (54-73)	63 (51-72)	68 (58-75)	60 (49-69)	57 (45-68)	61 (49-72)
Female	64%	58%	79%	79%	77%	78%	63%	64%
Disease duration, median years (IQR)	9.1 (4.5-15.0)	9.9 (5.0-15.9)	12.5 (6.8-17.9)	12.3 (7.3-18.0)	14.2 (9.2-18.8)	11.0 (5.8-17.1)	10.5 (5.4-16.5)	N/A
DAS28, median (IQR)	2.6 (1.9-3.4)	2.4 (1.7-3.4)	3.0 (2.3-4.2)	2.0 (1.1-3.3)	2.8 (2.0-3.8)	3.2 (2.4-4.4)	2.5 (1.8-3.6)	N/A
Missing DAS28	82%	72%	69%	71%	63%	63%	71%	N/A
Number of previous b/tsDMARDs, median (IQR)	0 (0-0)	0 (0-1)	1 (0-2)	0 (0-2)	0 (0-1)	2 (0-3)	0 (0-1)	N/A
Other b/tsDMARD use in 180 days prior to entry	1%	4%	10%	9%	6%	15%	6%	N/A
Concomitant csDMARD use	100%	37%	43%	31%	38%	32%	37%	1%
Concomitant steroid use	20%	14%	37%	28%	31%	30%	18%	2%
History of comorbidity*								
Cancer	5%	1%	3%	2%	5%	2%	2%	3%
Diabetes	14%	9%	15%	12%	15%	12%	10%	10%
Heart failure	3%	1%	4%	1%	4%	2%	2%	2%
Ischemic heart disease	6%	3%	7%	3%	7%	4%	4%	3%
Infection	5%	3%	8%	4%	8%	7%	4%	2%
Lung disease	9%	6%	17%	10%	18%	11%	8%	5%
Kidney failure	3%	2%	4%	2%	4%	2%	2%	2%
Stroke	4%	2%	3%	2%	3%	2%	2%	2%
Surgery	14%	12%	24%	24%	25%	21%	14%	6%
VTE	1%	1%	1%	1%	1%	1%	1%	1%
Highest education achieved								

<9 years	14%	5%	10%	8%	11%	7%	6%	9%
10-12 years	58%	58%	59%	57%	60%	60%	58%	55%
12+ years	28%	37%	31%	34%	30%	33%	36%	37%
Missing	1%	0%	0%	0%	1%	0%	0%	1%
Married/partner (%)	51%	49%	52%	50%	49%	50%	49%	48%
Born in Sweden (%)	90%	90%	90%	90%	86%	88%	89%	87%
Missing country of birth (%)	2%	2%	2%	2%	2%	2%	2%	3%

*See Supplementary Table S4 for definitions
csDMARD= conventional synthetic disease modifying anti-rheumatic drug, b/tsDMARD= biological/targeted disease modifying anti-rheumatic drug, DAS28=disease activity score on 28 joints, JAKi= Janus kinase inhibitors, N/A=not applicable, TNFi= tumor necrosis factor inhibitor, VTE=veneous thromboetic event

Table S7: Basic descriptive statistics of inflammatory joint diseases at risk during the years 2015-2020 in Sweden

	**Year**					
	**2015**	**2016**	**2017**	**2018**	**2019**	**2020**
N	96601	100274	103927	107488	111585	115317
RA	52%	51%	50%	49%	49%	48%
Female	62%	62%	62%	61%	61%	61%
Age at entry	61 (48-71)	61 (48-71)	61 (48-72)	61 (48-72)	62 (48-72)	62 (48-73)
Disease duration	7.6 (3.7-11.8)	8.0 (3.9-12.6)	8.5 (4.2-13.2)	8.9 (4.5-13.9)	9.3 (4.6-14.6)	9.7 (4.8-15.2)

Table S8: Treatment dispensations of all inflammatory joint diseases over years 2015-2020 in Sweden presented as the percentage of unique number of patients across each year

	**Year**					
	**2015**	**2016**	**2017**	**2018**	**2019**	**2020**
**Individuals, n**	96 601	100 274	103 927	107 488	111 585	115 317
**Treatment, %**						
**b/tsDMARDs**						
TNFi						
Adalimumab	29.7	29.5	26.7	26.3	35.2	38.0
Certolizumab pegol	7.7	7.2	6.7	6.8	6.0	5.3
Golimumab	15.4	14.6	12.6	11.4	9.6	8.2
Etanercept	43.2	47.9	56.6	64.2	57.6	46.0
Abatacept	4.7	5.3	5.9	6.7	7.1	8.0
IL-inhibitors						
Anakinra	0.6	0.6	0.5	0.4	0.6	0.7
Sarilumab				0.2	0.7	1.1
Tocilizumab	3.1	4.2	5.0	4.8	4.5	5.5
JAKi						
Tofacitinib	0.0	0.0	0.4	1.0	2.0	2.4
Baricitinib			0.5	3.5	5.1	5.1
Upadacitinib						0.3
Apremilast	0.5	1.9	1.9	2.0	2.0	1.8
**csDMARDs**						
Sulfasalazine	31.8	30.6	29.9	28.0	27.1	24.5
Methotrexate	132.7	131.8	129.3	126.8	123.9	116.3
Hydroxychloroquine	9.1	9.4	9.7	9.6	9.3	9.4
Leflunomide	4.4	4.4	4.1	4.0	3.9	3.8
**Other**						
Prednisolone	130.6	128.5	126.2	124.9	123.6	118.1
Guselkumab				0.0	0.1	0.2
Secukinumab	0.1	1.7	3.4	4.4	4.8	5.0

Ixekizumab			0.1	0.2	0.5	0.8

TNFi= tumor necrosis factor inhibitor, JAKi= Janus kinase inhibitors, IJD=inflammatory joint disease

Percentages here represent the number of starts per treatment divided by the number of individuals

Figure S1: Monthly incidence proportion of rheumatoid arthritis and of a combined group of all inflammatory joint diseases in Sweden during 2015-2020. Bold lines represent the beginning of the COVID-19 pandemic.

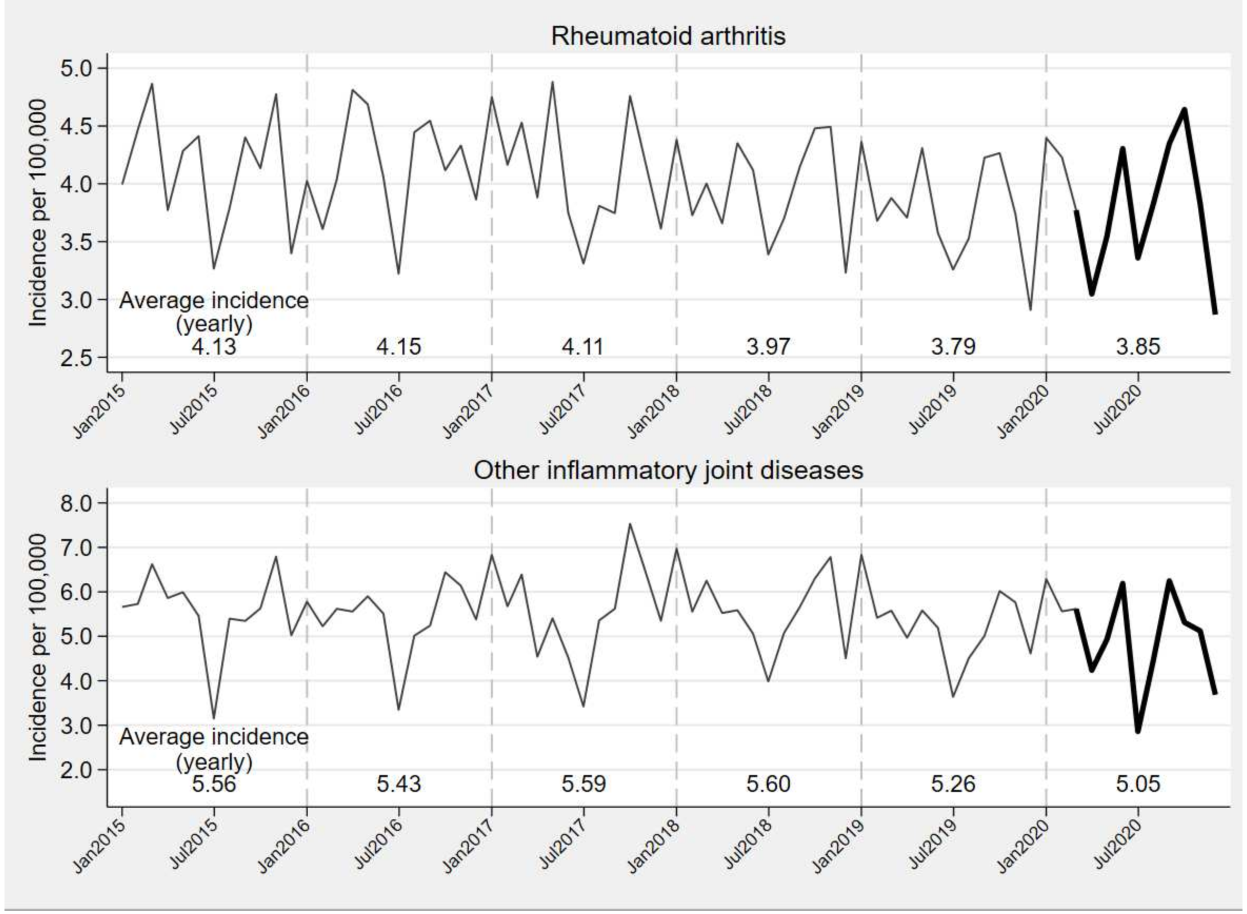

Figure S2: Percentage of visits in 2020 (N visits/individuals) compared to the average during 2015-2019 for inflammatory joint disease patients in Sweden

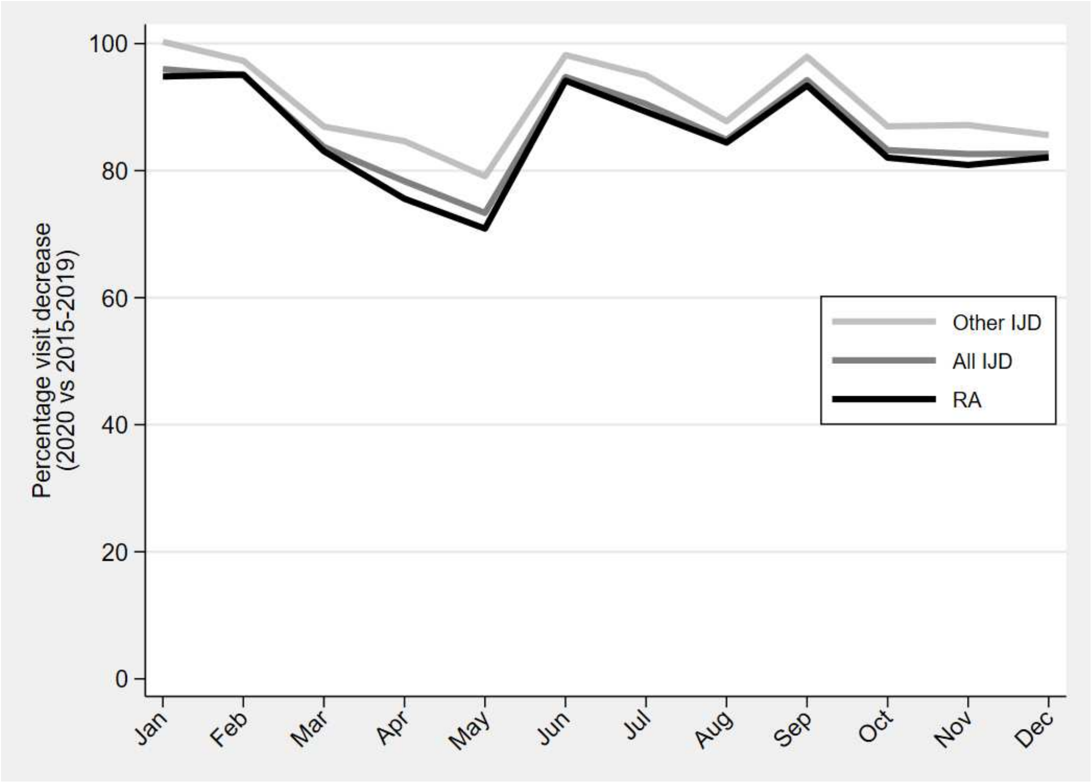

Figure S3: Average number of visits to rheumatology or internal medicine listing ICD-10 diagnosis codes for inflammatory joint disease per month (total number of visits/patients alive at beginning of each month) for patients with rheumatoid arthritis (RA) and other inflammatory joint diseases (IJD) in Sweden between 2015 and 2020

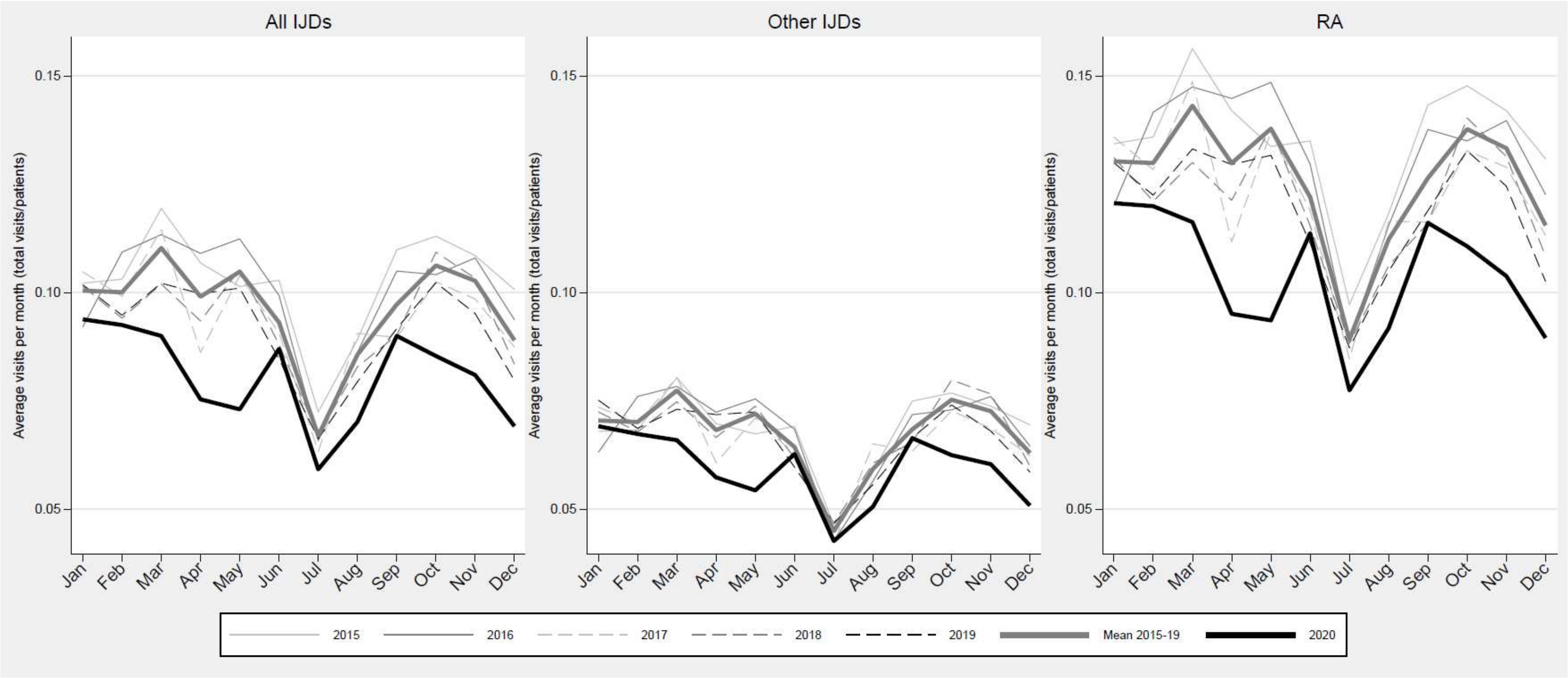

Figure S4: Average number of visits for patients with all inflammatory joint diseases in Sweden between 2015 and 2020, presented by disease duration

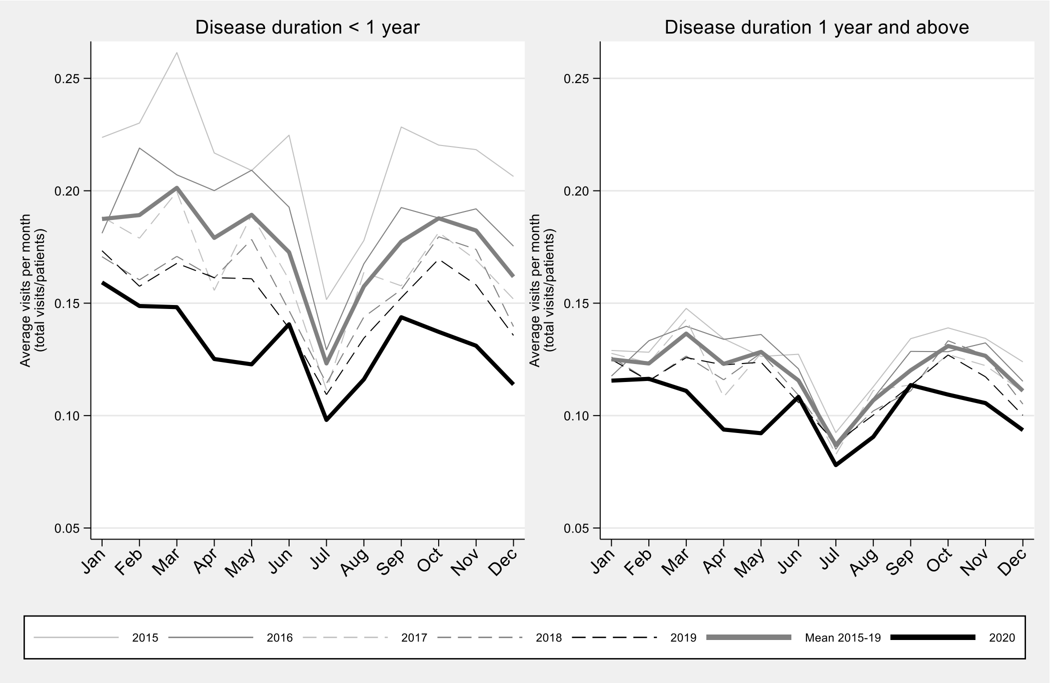

Figure S5: Average number of visits for patients with all inflammatory joint diseases in Sweden between 2015 and 2020, presented by age

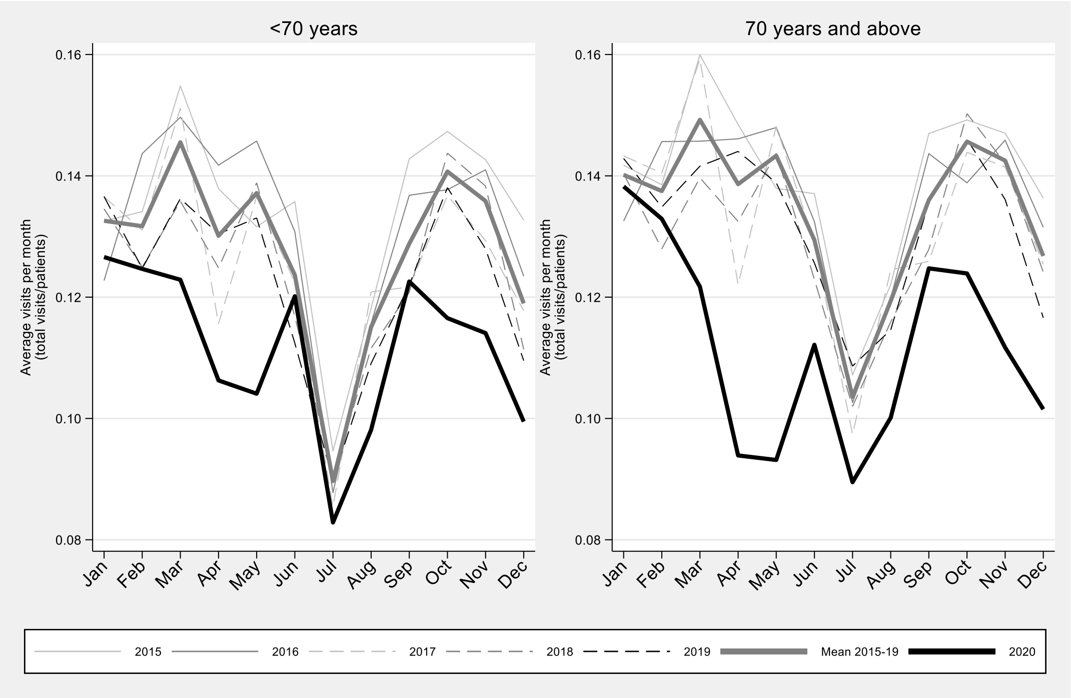

Supplement: Supplementary data [file rmdopen-2021-001987supp001.pdf]
